# Development and feasibility testing of a training programme for community pharmacists to deliver a culturally responsive medication review intervention

**DOI:** 10.1186/s40814-022-01006-2

**Published:** 2022-03-03

**Authors:** Amanda J. Wheeler, Jie Hu, Santosh Kumar Tadakamadla, Kerry Hall, Adrian Miller, Fiona Kelly

**Affiliations:** 1 Menzies Health Institute Queensland, Griffith University, Nathan campus, Brisbane, 4111 Australia; 2 Faculty of Medical and Health Sciences, University of Auckland, Auckland, New Zealand; 3 CQ University, Townsville, Qld Australia; 4 School of Pharmacy & Medical Sciences, Griffith University, Gold Coast, Australia

**Keywords:** Aboriginal and Torres Strait Islander people, Indigenous health, Feasibility study, Pilot study, Community pharmacists, Cultural training, Medication

## Abstract

**Background:**

Cultural differences between health professionals and Indigenous peoples contribute to health inequalities, and effective cross-cultural communication and person-centred healthcare are critical remedial elements. Community pharmacists can play a significant role by reducing medication-related problems through medication reviews, yet barriers to access include cultural and linguistic challenges. The Indigenous Medication Review Service (IMeRSe) aimed to address these barriers via a culturally responsive intervention. The aim of this paper is to present the cross-cultural training framework developed as a component of this intervention and the feasibility evaluation of the first stage of the training framework.

**Methods:**

A training framework was developed, emphasising pharmacists’ skills and confidence in effective cross-cultural communication and relationship-building with Indigenous Australians (Please note that the use of the term ‘Indigenous’ in this manuscript includes all Aboriginal and Torres Strait Islander people and acknowledges their rich traditions and heterogenous cultures) across three stages: (1) online and workshop-based, covering Indigenous history and health, cross-cultural communication and a holistic, strengths-based approach to intervention delivery; (2) orientation to local Aboriginal Health Services, community and cultural protocols; and (3) ongoing mentoring. The feasibility evaluation of the first stage included the following: self-reported levels of cultural capability, cultural confidence and skills, motivators and barriers to working with Indigenous Australians, assessed pre- and post-training. Participants completed self-administered questionnaires including a 22-item validated Cultural Capability Measurement Tool. Paired *t* tests assessed change in mean scores of Likert scale data.

**Results:**

Stage 1 development resulted in an 8.5-h standardised cross-cultural training programme tested with 39 pharmacists working across urban and rural/remote Australia. Thirty-six pharmacists completed the feasibility evaluation (75.7% female, all non-Indigenous, 75.7% never attended prior cross-cultural training). Participants reported overall acceptability with training; the majority perceived it added value to their practice. Improved cultural capability post-training was reflected in increased scores for 21/22 items, nine reaching statistical significance. There were significant improvements for all 26 confidence and skills statements, and selected motivational and barrier statements, particularly participants role in improving Indigenous health outcomes and cross-cultural communication.

**Conclusions:**

This study provides preliminary evidence that the training programme was feasible to deliver and prepared pharmacists to deliver a culturally responsive medication review intervention. The online knowledge-based modules and face-to-face workshops provide a standardised framework for larger-scale implementation of the intervention training.

**Trial registration:**

Australia and New Zealand Clinical Trials Registry ACTRN12618000188235.Prospectively registered 22 January 2018.

## Key messages re feasibility

- Community pharmacists can play a significant role by reducing medication-related problems through medication reviews, yet barriers to access include cultural and linguistic challenges. The Indigenous Medication Review Service (IMeRSe) aimed to address these barriers via a culturally responsive intervention. A training framework for pharmacists was identified as an essential component of the intervention which required development and evaluation.
- This study provides preliminary evidence that the cross-cultural training programme developed as an essential component of the IMeRSe study was acceptable and feasible, preparing community pharmacists to facilitate delivery of a culturally responsive medication review intervention for Indigenous Australians in urban centres or remote communities.
- The multifaceted training programme included a standardised online module and face-to-face workshop delivered alongside a training manual which can be operationalised for larger-scale implementation of the intervention. This study provides support for cross-cultural training, as a critical element in the implementation of culturally safe health services for Indigenous people.

## Background

Wide disparities in health outcomes between Aboriginal and Torres Strait Islander people (hereafter, respectfully referred to collectively as Indigenous Australians)^1^, and non-indigenous Australians are well documented, and whilst there have been areas of improvement, including life expectancy, inequities persist [1]. Social and cultural differences between health care providers and Indigenous people are recognised as contributing to the ongoing health inequalities resulting in higher rates of illness and hospitalisations [2, 3]. Effective cross-cultural clinical interactions and person-centred care delivery require health professionals with the knowledge, skills and confidence to manage these encounters more successfully and, hence, reduce health disparities.

Providing cross-cultural education and training for the health workforce has increased in Australia over the last decade [4, 5]. In 2014, the Department of Health published the Aboriginal and Torres Strait Islander Health Curriculum Framework, enabling higher education providers to train and graduate health professionals equipped to provide culturally safe health services for Indigenous peoples [5]. The framework is supported by the principles of Indigenous Allied Health Australia (IAHA); emphasising the need for a rights-based, culturally responsive, approach that is holistic, evidence-driven, strengths-based and inclusive of diversity between and within communities [6].

Cross-cultural education and training (often referred to interchangeably with related terms such as cultural awareness, cultural safety, cultural competence, cultural intelligence and/or cultural capability education/training) has been shown to improve the attitudes, knowledge, confidence and skills of health care providers and to improve patient satisfaction and health care access [7, 8]. A 2018 systematic review of health workforce cultural competence interventions suggested health outcomes could be improved by prioritising practical skills development in training and monitoring the consistent application of these skills in healthcare encounters [7]. A later study similarly found that a single workshop could be effective in building health professionals confidence in providing culturally safe care for Indigenous Australians and that this improvement was sustained at two months [9].

Disparities in health outcomes between Indigenous and non-indigenous Australians are particularly apparent amongst Indigenous Australians living with chronic diseases. In 2016, nearly three in four Indigenous deaths resulted from diseases including cardiovascular, respiratory, cancer and diabetes, and these diseases accounted for almost 80% of the difference in mortality between Indigenous and non-Indigenous Australians [10]. Community pharmacists play a significant role in chronic disease management through pharmacy programmes such as medication review services [11]. Pharmacist-led medication review services, funded by the Australian government, include MedsCheck/Diabetes MedsCheck,^2^ and Home Medicines Review (HMR)^3^. Both services have shown benefits in reducing medication-related problems, a direct consequence of which is potentially preventable hospitalisations amongst Australians with chronic diseases [11]. Despite the recognised benefit from these medication review services, particularly for those with chronic conditions and after discharge from hospital [12, 13], they are not well utilised by Indigenous Australians [14, 15]. Research has indicated that the current service delivery model does not meet the needs of this population and that a culturally responsive service is urgently needed [16–19]. Barriers to success have been identified and include cultural and linguistic challenges, geographical isolation, lack of involvement by Indigenous health staff and the one-off, short-term focus of medication reviews despite the long-term nature of these health conditions. Furthermore, the continuing legacy of colonisation has contributed to a lack of trust in health services and non-Indigenous staff. To help ameliorate these barriers, Indigenous health workers often accompany patients to ensure cultural ‘translation’ and facilitate appropriate encounters.

In response to calls for a medication management service that prevented medication-related problems, and that was flexible and responsive to the needs of Indigenous people [19–22], the Australian Government funded the Indigenous Medication Review Service feasibility study (IMeRSe) in 2017. The IMeRSe intervention aimed to optimise individual Indigenous consumers medication management, delivered by community pharmacists integrated with Aboriginal health services [23]. A training framework for pharmacists was identified as an essential component of the intervention which required development and evaluation [24]. The framework focused on building/strengthening interpersonal and interprofessional relationships for pharmacy and Aboriginal health service (AHS) staff and for pharmacists to develop the skills and confidence necessary to promote effective communication and relationship building with Indigenous consumers. The framework involved three stages:

1. Cultural responsiveness, communication, and a holistic, strengths-based approach to intervention delivery that was suitable for larger-scale implementation. The development and evaluation of this stage is described in this paper;
2. Introduction, welcome and orientation to the local AHS and their community. This stage was facilitated by local AHS staff and provided local and regionally specific information, to promote a shared understanding of the history of that community and their cultural protocols;
3. Mentoring for pharmacists during the active intervention delivery period (2018–2020) to address the specific health and cultural needs of Indigenous participants and their communities. This stage was led by a mentor pair who were experienced clinicians and academic educators (a pharmacist; and an Indigenous health practitioner). The latter two stages, not described in this paper, are part of the larger IMeRSe effectiveness and cost-effectiveness evaluation.

This paper reports on the first training stage; the development and feasibility evaluation of a cross-cultural training programme for community pharmacists to facilitate delivery of a culturally responsive medication review intervention for Indigenous Australians.

## Methods

### Context

The Indigenous Medication Review Service feasibility study (IMeRSe) was established as a partnership project involving the Pharmacy Guild of Australia, the National Aboriginal Community Controlled Health Organisation (NACCHO) and Griffith University (see www.griffith.edu.au/imerse). An Expert Panel (including Indigenous advisors) was established at the outset to provide guidance to the research team throughout the study, including commentary on the development and piloting of training materials and resources. Following consultation with the Expert Panel, nine AHSs and 25 associated community pharmacies from three of the eight Australian states/territories enrolled in the study. Ethics approval for the study was obtained from the relevant agencies including the Griffith University Human Research Ethics Committee who approved both the training pilot (2018/187) and the final training programme for evaluation (2018/251). Written informed consent was obtained from the owners/managers of all community pharmacies participating in the feasibility study, and from each pharmacist participating in the training and its evaluation. The protocol for the feasibility study has been published elsewhere [23]; it outlines the study outcomes which will evaluate the effectiveness and cost-effectiveness of the IMeRSe intervention. The study was registered (ACTRN12618000188235).

### Development of the training programme

The content of the programme, learning techniques and resource materials were informed/confirmed by reviewing key documents and consultations with experts in the field (Table 1).

**Table 1:** Consultation and resources reviewed in the training development process

Key resources	Experts involved^a^
- National Continuous Quality Improvement Framework for the ACCHS Seamless Client/Patient Journey [25] - National Aboriginal And Torres Strait Islander Health Plan 2013-2023 [26] - National Medicines Policy [27] - National key performance indicators for Aboriginal and Torres Strait Islander Primary Health Care [28] - Guide to providing pharmacy services to Aboriginal and Torres Strait Islander people developed as part of the 5^th^ CPA [29] - Interpretive guide to the RACGP Standards for Aboriginal community controlled health services [30] - Cultural Responsiveness in Action; Aboriginal and Torres Strait Islander Allied Health Australia (IAHA) Framework [6] - Aboriginal and Torres Strait Islander Health Curriculum Framework [5] - Reconciliation Australia’s website [31]	- Two senior Indigenous researchers with expertise in health delivery for Indigenous people; - Three Indigenous health practitioners and academics with experience training cultural awareness with health students and professionals; - One Indigenous trainer of cultural awareness across the retail and corporate sectors for ten years; - Two representatives of NACCHO; - A GU academic with expertise in developing and delivering training to practicing pharmacists and students; - An academic with expertise in pharmacy services and in medication management in Indigenous people; - A GU pharmacy academic with expertise in person-centred care and innovative health service implementation strategies in community pharmacies; - A GU academic and pharmacist accredited in medication reviews (20 years); - Seven pharmacists, all with experience in pharmacy services, three with experience working in Indigenous Health; - One Indigenous representative from a state health organisation; and - Two doctors with experience in working and researching in Indigenous health.

*GU* Griffith University, NACCHO National Aboriginal Community Controlled Health Organisation ^a^Note that some experts provided expertise from multiple perspectives, e.g. academic with research experience in Indigenous Health and experience in medication reviews as an accredited pharmacist

The first stage of training comprised an online module which participants completed prior to attending a one-day, face-to-face, workshop.

The online module consisted of four components encouraging participants to reflect on their own culture and cross-cultural communication through insights into Indigenous health and history; it required approximately 1.5 h to complete. The components included the following: (i) *Understanding history & culture* provided insights into the history and culture of Indigenous Australians, including reconciliation (via www.shareourpride.org.au/);( ii) *Australian context and Indigenous health* explained the persistent and ongoing nature of health inequalities, utilising interactive health maps; (iii) *Pharmacy context* provided information on pharmacy services available for Indigenous people; and (iv) *Important strategies and resources* introduced participants to teminology and key intercultural communication strategies including videos used previously to support relationship building with Indigenous Australians in health service delivery. In addition, post-training resources were provided to encourage ongoing reflection on culture, communication using appropriate terminology and cultural protocols. The online module was made available to participants as an adaptive lesson hosted by Smart Sparrow (www.smartsparrow.com).

Participants were provided with overall learning objectives for both parts of the cross-cultural training programme and specific objectives for the online module to benchmark knowledge expectations at the face-to-face workshop. To promote a reflective approach to learning, participants were asked to identify their own learning needs at the start of the online module, and then on completion of each of the four components, reflect on what they had learnt and how they would apply it in practice, and to identify further learning needs. For the *Pharmacy context* component (iii), pharmacists were provided with published guidelines and case examples and asked to reflect on these materials online, in the context of their practice experience.

The second part, face-to-face training, delivered as a 1-day workshop was interactive and built on the online content. The workshop comprised two major components delivered by a four person training team, two of whom were Indigenous Australians. Modules included the following: Indigenous history, culture, and health (Module 1) and medication management including intervention delivery (Modules 2–6). Module 1 was delivered by an experienced Indigenous training partnership, an educator and an Aboriginal health practitioner. Modules 2–6 were presented by a pharmacist training partnership; pharmacist educators experienced in delivering innovative pharmacy service implementation strategies and medication review services. The educators were supported by the Aboriginal health practitioner.

The workshop focussed on delivering a strengths-based, collaborative intervention demonstrating effective cross-cultural communication strategies for building relationships with AHS staff and consumer participants. An Indigenous-specific tool integral to the intervention was the *Stay Strong App* [32], which formed the basis of the medication review conversation (called the *Medicines Talk*). The four-step App was initially developed as an e-mental health resource for use with Indigenous Australians. It used a holistic, strengths-based approach, which combined therapeutic problem-solving and motivational interviewing, in a visually-appealing interface. The *Stay Strong App* positions the health worker and patient as collaborative partners and faciltates an exploration of the patient’s family and important cultural connections, associated strengths, worries and goals for change. The App was selected for the IMeRSe feasibility study as a conduit to increase pharmacists confidence when talking with Indigenous participants and their families about their health and well-being concerns, and their future health-related goals [32, 33].

A combination of delivery styles and formats was used in the one-day workshop, including Microsoft PowerPoint presentations and discussion, group activities, case-vignettes and role plays based on real-life scenarios. Example communication interactions between simulated patients (Indigenous actors), pharmacists and Indigenous health workers using the *Stay Strong App* in real-time, were filmed as case-vignettes. Training participants watched and critiqued the pre-recorded footage and were encouraged to share their own experiences and to role-play using the *Stay Strong App* as a tool to initiate the medication review conversation (*Medicines Talk*) and to build relationships*.* Experiential learning techniques, such as real-life scenarios and role-plays, were incorporated into the training to raise awareness of cultural and communication issues, and to provide participants with opportunities to interact with the training materials, which has been shown to improve understanding and confidence [34]. All of the training materials, activities, pre-recorded case-vignette examples and the *Stay Strong* tool were included in a training manual provided to participants.

The training was piloted with eight, final year Master of Pharmacy students, six registered pharmacists (including academics involved in implementing and evaluating new pharmacy services, medication management experts, pharmacists working in Indigenous health and two researchers with expertise in health inequalities, particularly Indigenous health). Qualitative assessment was obtained at the completion of the pilot workshop through two focus groups conducted by experienced facilitators. Recommended changes were made to the training content, for example, content on region-specific cultural history relevant to the study sites was added to the online module, and more culturally responsive communication strategies and opportunities for role-play were included in the workshops.

Finally, the training content was confirmed as aligning with the principles of the Aboriginal and Torres Strait Islander Health Curriculum Framework [5] and the core principles of IAHA [6].

### Feasibility evaluation of the training

#### Study design and participants

A pre-post assessment design was used to evaluate the first stage of training. Self-administered online questionnaires^4^ were completed by participants before commencing the online training (T1) and after completion of the face-to-face workshop (T2). Four face-to-face workshops were conducted in May, July, September and November 2018. All participants were provided with access to the online component approximately 2–3 weeks before the face-to-face workshop. Each workshop was delivered in accordance with the aforementioned training manual. Participants were pharmacists from the 25 community pharmacies enrolled in the IMeRSe feasibility study.

#### Outcome measures

The T1 questionnaire collected participants characteristics. Both T1 and T2 questionnaires included a validated measure: the Cultural Capability Measurement Tool [35, 36] and questions developed or adapted from other studies [37–39] to self-evaluate confidence, skills, motivations and barriers to working with Indigenous people.

The Cultural Capability Measurement Tool (CCMT) has 22 items representing five core cultural capabilities (respect, communication, safety and quality, reflection, and advocacy) that are reflected in the Aboriginal and Torres Strait Islander Health Curriculum Framework [5].

- *Respect* refers to participants acknowledging Indigenous ways of knowing, being and doing in the context of history, culture and diversity;
- *Communication* refers to the importance of culturally responsive communication in professional practice;
- *Safety and quality* refers to participants’ capabilities to apply high-quality evidence and strengths-based approaches when delivering care to Indigenous people;
- *Reflection* refers to participants ability to reflect on their own culture, professional culture and dominant cultural paradigms;
- *Advocacy* overlaps with *Reflection* to some degree and refers to participants’ capability to engage in cultural change [5].

Responses to items were rated on a Likert scale, 1 = *strongly disagree* to 5 = *strongly agree*, whilst responses to 14 items were reverse coded, i.e. *strongly agree* = 1 to *strongly disagree* = 5. A higher score indicated stronger cultural capability.

Confidence and skills in working with Indigenous people were assessed using a set of 26 statements, also using a 5-point Likert scale. Motivations and barriers to working with Indigenous people were assessed using a series of 8 and 10 statements respectively, with responses similarly rated on a 5-point Likert scale. These statements were developed and adapted from existing research as part of the review and consultation process outlined in Table 1 and particularly as they related to pharmacy practice change [37–39].

Acceptability of the training was assessed by additional quantitative and qualitative questions in the T2 questionnaire which related to the learning content, presentation and delivery of the training.

Questionnaires used Likert-type rating scales (e.g. from 1 = *strongly disagree* to 5 = *strongly agree*) and multiple-choice options, with some free-text responses.

Both the T1 and T2 questionnaires were assessed for face validity as part of the training pilot. Minor amendments were made to the wording of a few questions, and some questions were deleted based on the feedback received. The final questionnaires each required approximately 20 min to complete online.

#### Data analysis

All data analyses were performed using Stata 13.1. Descriptive statistics were employed to describe participants’ demographic characteristics and report on participants’ acceptability with respect to the training. Paired *t* tests were conducted to test the change in mean scores of the Likert scale data between T1 and T2. A *p*-value of less than 0.05 was considered as statistically significant.

## Results

Thirty-nine participants completed the online module and attended a workshop; 37 completed the pre-and post-training questionnaires respectively; however, matching pre-post assessment was only available for 36 participants. Participants characteristics are presented in Table 2: the majority were female (28/37, 75.7%) and spoke English as their primary language at home (30/37, 81.1%); all were non-indigenous Australians, and three quarters (28/37, 75.7%) had never previously attended any form of cross-cultural training. Fifteen participants were born overseas, although most (13/15) had lived in Australia for more than 10 years. Pharmacy practice experience ranged from less than a year to 30 years, with 13 participants reporting more than 10 years.

**Table 2:** Demographic characteristics of study population (*n* = 37  T1 participants)

	*n* (% )
Gender	
Male	9 (24.3)
Female	28 (75.7)
Age^a^	
< =30	19 (52.8)
> 30	17 (47.2)
Country of birth	
Australia	22 (59.5)
Other^b^	15 (40.5)
Years lived in Australia (if not born in Australia, *n* = 15)	
6-10 years	2 (13.3)
> 10 years	13 (86.7)
Language spoken at home	
English	30 (81.1)
Other^c^	7 (18.9)
Highest qualification	
Bachelor degree	30 (81.1)
Masters/PhD degree	7 (18.9)
Attended cultural awareness training in the past	
Yes	9 (24.3)
No	28 (75.7)
Community pharmacy experience^a^	
0–10	22 (62.9)
11–20	8 (22.9)
> 20	5 (14.2)

^a^Total number not equal to 37 due to missing responses

^b^Other includes New Zealand, USA, Vietnam, Singapore, Papua New Guinea, India and Philippines

^c^Other includes Vietnamese, Sinhala, and Malayalam

### Acceptability

**Fig. 1:**
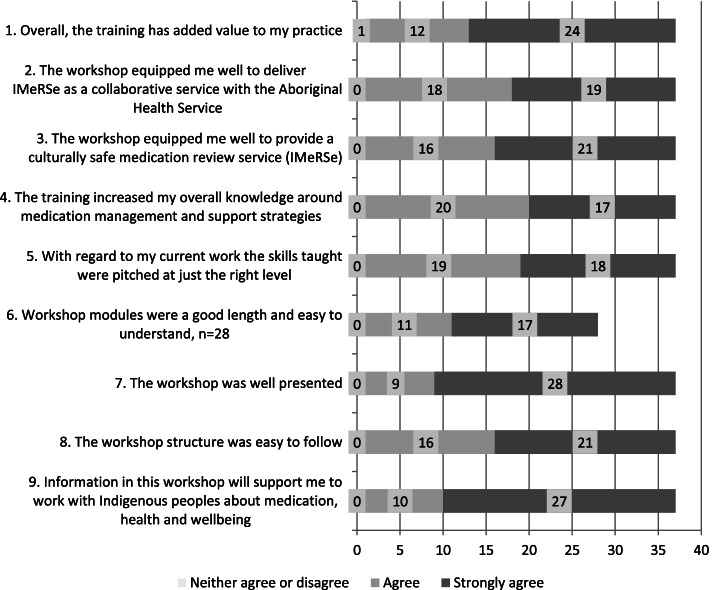
Training acceptability (*n* = 37  T2 participants)

Participants reported overall acceptability with the training learning content, delivery and structure, that they would recommend the training to other pharmacists, with the majority perceiving that it had added value to their current practice (Fig. 1). Qualitative feedback included:

“It [the training] was really good. I went in knowing next to nothing to be honest and came out, I think I can do this. So that’s great.” [P1F]

“I think the training, the face–to-face training…was very helpful for me in the way it helped me improve my communication with the client, when I conduct the interview [Medicines Talk] with them.” [P4M]

“…the training, was about finding ways to make the patient comfortable having these conversations with us. I think …after the training, I feel a lot more confident in being able to do this.” [P1F]

Areas of the training that participants reported were least useful or could be improved included: not having enough time in the workshop to practice knowledge and skills (69.4%), wanting more resources (36.1%) and a longer workshop (33.3%). Some participants (36.1%) reported the need for further refresher training and ongoing support during the study implementation, such as more real-life scenarios/practice. However, participants also reported that the most useful aspects of training were the motivating workshop presenters (77.8%) and their access to ongoing support from mentors to implement their knowledge and skills in practice (55.6%).

All participants stated that they would recommend the training for other pharmacists.

### Quantitative feasibility measures

#### Cultural capability

Table 3 summarises pre-post assessment in cultural capability using the 22-item CCMT. Mean scores were significantly higher at T2 for nine items indicating improved cultural capability. Whilst there were increases in mean scores for another 12 items, this change was not statistically significant. One item “I feel comfortable working with people from other cultures” had a high mean score of 4.26 at T1, and this did not change at T2.

**Table 3:** Self-reported assessment of cultural capability (Cultural Capability Measurement Tool) (*n* = 36)

	n*	T1 mean (SD)	T2 mean (SD)	***p***
History does not impact on First Peoples health ^a^	35	4.11 (0.76)	4.40 (0.98)	**0.023**
Understanding First Peoples social practices will not apply to my practice ^a^	35	4.37 (0.65)	4.57 (0.56)	0.090
I find it difficult to understand the beliefs of different cultural groups ^a^	35	3.97 (0.66)	4.37 (0.60)	**0.006**
Reflecting on my own cultural values will not help me become culturally aware ^a^	35	4.09 (0.82)	4.46 (0.56)	**0.017**
Improving First Peoples health is not the responsibility of all health professionals ^a^	35	4.48 (0.51)	4.69 (0.47)	0.051
It is not my responsibility to challenge the way things are done in health practice ^a^	35	4.29 (0.79)	4.40 (0.77)	0.554
My relationship with First Peoples will not impact on clinical outcomes ^a^	35	4.23 (0.84)	4.54 (0.51)	**0.020**
All First Peoples are treated equally by health professionals ^a^	35	3.23 (1.24)	4.00 (0.77)	**< 0.001**
First Peoples receive special treatment from government ^a^	35	2.71 (0.89)	3.83 (1.01)	**< 0.001**
First Peoples have the same level access to health services as all other Australians ^a^	35	3.43 (0.92)	3.97 (0.95)	**0.005**
I will find it difficult to advocate for improvements in First Peoples health ^a^	35	4.03 (0.75)	4.17 (0.71)	0.324
It is difficult for me to be culturally inclusive towards First Peoples ^a^	35	4.23 (0.73)	4.43 (0.61)	0.109
I do not have a social responsibility to work for changes in First Peoples health ^a^	35	4.29 (0.52)	4.46 (0.66)	0.160
There may be few exceptions but in general First Peoples are all the same ^a^	33	3.91 (0.88)	4.00 (0.97)	0.540
Understanding First Peoples history will inform my practice as a health professional	35	4.14 (0.81)	4.51 (0.51)	**0.014**
Understanding First Peoples cultural values will influence how I practice	35	4.20 (0.72)	4.43 (0.65)	0.103
To improve First Peoples health, Indigenous cultures need to be visible in clinical and community health settings	35	4.09 (0.70)	4.37 (0.73)	**0.023**
I feel comfortable working with people from other cultures	35	4.26 (0.70)	4.26 (0.61)	1.00
Acknowledging that cultural differences exist is the first step to becoming culturally capable	35	4.23 (0.97)	4.51 (0.56)	0.152
Comprehensive primary health care services are fundamental to improving First Peoples health	35	4.46 (0.78)	4.51 (0.56)	0.711
Evidence from research can help me in my practice in First Peoples health	35	4.26 (1.02)	4.41 (0.56)	0.419
I believe a holistic approach to First Peoples health is important	35	4.40 (0.91)	4.54 (0.51)	0.361

* Not all participants answered each question

^a^ Reverse-coded items: 1 = strongly agree to 5 = strongly disagree; all other items were coded as 1 = strongly disagree to 5 = strongly agree

*SD* standard deviation; *p* < 0.05 indicates statistical significance. Higher score indicates better cultural capability

#### Cultural confidence and skills

Comparison of mean scores for 26 confidence and skills statements at T1 and T2 are presented in Table 4. At T2, participants had significantly higher scores for all 26 statements indicating improvements in both areas.

**Table 4:** Self-reported assessment of confidence and skills (*n* = 36)

	***n***^**a**^	T1 mean (SD)	T2 mean (SD)	***p***
I am confident in communicating with Aboriginal and Torres Strait Islander people about health and wellbeing concerns	35	3.69 (0.72)	4.00 (0.59)	**0.026**
I am confident in using a culturally sensitive approach to talk with Aboriginal and Torres Strait Islander people about their medication	35	3.23 (0.88)	3.94 (0.64)	**< 0.001**
I am confident that I know enough about cultural safety to carry out my pharmacist role when working with Aboriginal and Torres Strait Islander people about their health problems	35	2.83 (0.95)	3.83 (0.75)	**< 0.001**
I feel confident discussing Aboriginal and Torres Strait Islander health-related issues for my consumer with their GP or other provider	35	3.74 (0.92)	4.26 (0.56)	**0.001**
I feel confident working with Aboriginal and Torres Strait Islander people to improve their understanding of and adherence to medications	35	3.77 (0.91)	4.11 (0.72)	**0.021**
I am confident of using a culturally sensitive approach when working with Aboriginal and Torres Strait Islander people about medication-related problems	35	3.06 (0.94)	4.06 (0.59)	**< 0.001**
I am confident in dealing with differences between my culture and the culture of Aboriginal and Torres Strait Islander peoples	35	3.29 (0.93)	4.06 (0.64)	**< 0.001**
I am confident in managing cross-cultural medication adherence issues	35	3.31 (0.76)	4.09 (0.61)	**< 0.001**
I feel confident to apologise for cross-cultural misunderstanding or errors	34	3.47 (0.99)	4.15 (0.70)	**< 0.001**
I am confident that I can be attentive to nonverbal cues or the use of culturally specific gestures that might have different meanings in different communities	35	3.20 (0.83)	4.03 (0.75)	**< 0.001**
I feel confident I can elicit individual perspectives about health and illness from Aboriginal and Torres Strait Islander people	35	3.26 (0.78)	3.89 (0.72)	**< 0.001**
I feel confident to work with a consumer who uses Bush medicine	35	2.43 (1.07)	2.91 (1.20)	**0.033**
I feel confident to incorporate culturally relevant information into a medication review service	35	3.06 (0.91)	3.77 (0.81)	**< 0.001**
I feel confident of being able to greet Aboriginal and Torres Strait Islander peoples in a culturally sensitive manner	35	3.31 (0.83)	3.94 (0.76)	**< 0.001**
I am confident in knowing how to ask a client if they are of Aboriginal and/or Torres Strait Islander descent	34	3.12 (0.95)	3.71 (1.00)	**0.012**
I feel confident in working with Aboriginal and Torres Strait Islander people on setting goals for medication adherence	35	3.49 (0.78)	4.06 (0.68)	**< 0.001**
I am confident in speaking to an Aboriginal and Torres Strait Islander consumer’s family/carers	35	3.71 (0.79)	4.17 (0.62)	**0.003**
I am confident in using medicine or health-related smart phone Apps to support people with their medication use	34	3.56 (0.82)	4.06 (0.74)	**0.009**
I am confident in knowing who or where to seek help from to support Aboriginal and Torres Strait Islander peoples	35	3.29 (1.13)	3.94 (0.76)	**0.002**
I feel confident in working with Aboriginal and Torres Strait Islander peoples on setting goals for lifestyle change	34	3.44 (0.93)	4.12 (0.69)	**< 0.001**
I know where to access culturally responsive resources for Aboriginal and Torres Strait Islander people with medication problems (e.g. written information and websites)	35	2.71 (0.96)	3.80 (0.72)	**< 0.001**
I have knowledge of the community and health services I can refer Aboriginal and Torres Strait Islander people to	35	3.54 (0.85)	4.09 (0.66)	**0.004**
I understand the concept of shame in the context of health for Aboriginal and Torres Strait Islander peoples	35	3.09 (1.12)	4.23 (0.69)	**< 0.001**
I understand the concept of embarrassment in the context of health for Aboriginal and Torres Strait Islander peoples	35	3.17 (1.07)	4.17 (0.66)	**< 0.001**
I understand the concept of a strengths-based approach to health and wellbeing	35	3.17 (1.10)	4.37 (0.55)	**< 0.001**
I think that cultural beliefs affect the health of Aboriginal and Torres Strait Islander peoples	35	4.06 (0.73)	4.49 (0.56)	**< 0.001**

^a^Not all participants answered each question. Higher scores indicate increased confidence and skills

#### Motivators and barriers

Participants showed significant improvements in four of eight motivational statements and four of ten barrier statements (Tables 5 and 6). Whilst there were positive changes in mean scores observed for all four other motivator statements and for five of the other six barrier statements, this change was not statistically significant. One statement “I do not have access to a suitable private area to talk with people” had a negative change at T2.

**Table 5:** Self-reported assessment of motivators (*n* = 36)

Motivators	***n***^**a**^	T1 mean (SD)	T2 mean (SD)	***p***
I am motivated to work with Aboriginal and Torres Strait Islander people and carers in my current role	35	4.37 (0.55)	4.51 (0.56)	0.096
Health and media campaigns, in general, have made society more concerned about the health of Aboriginal and Torres Strait Islander people	35	3.63 (0.69)	3.71 (0.83)	0.521
Aboriginal and Torres Strait Islander people request my advice about medicines	35	3.57 (0.78)	3.66 (1.03)	0.585
There are funding initiatives to support me in providing services for Aboriginal and Torres Strait Islander people	35	3.43 (0.78)	3.97 (0.86)	**< 0.001**
There is good evidence that pharmacists can improve health outcomes for Aboriginal and Torres Strait Islander people	35	3.86 (0.81)	4.34 (0.73)	**< 0.001**
Pharmacy professional bodies recognise pharmacist role in improving outcomes for Aboriginal and Torres Strait Islander people	35	3.66 (0.76)	4.26 (0.61)	**< 0.001**
Community pharmacists are an integral member of the health care team working with Aboriginal and Torres Strait Islander people	35	4.26 (0.61)	4.57 (0.56)	**< 0.001**
Community pharmacists should have a greater involvement with the health care team from Aboriginal Health Services	35	4.31 (0.68)	4.46 (0.56)	0.201

^a^ Not all participants answered each question. Higher scores indicate greater motivation

**Table 6:** Self-reported assessment of barriers (*n* = 36)

Barriers	***n***^**a**^	T1 mean (SD)	T2 mean (SD)	***p***
I find it difficult to work with Aboriginal and Torres Strait Islander consumers and carers in my present role	34	2.09 (0.79)	1.88 (0.73)	0.109
I am too busy dealing with the problems that other people present with	34	1.79 (0.69)	1.76 (0.70)	0.801
There are no appropriate self-help or educational pamphlets available to me	33	2.79 (0.99)	2.24 (0.83)	**0.012**
I do not have access to a suitable private area to talk with people	34	1.76 (0.89)	1.91 (1.06)	0.257
I do not have access to enough consumer clinical information	33	2.09 (0.95)	1.76 (0.71)	0.110
I do not know how to talk to Aboriginal and Torres Strait Islander people	34	2.18 (0.94)	1.76 (0.65)	**0.006**
Working with Aboriginal and Torres Strait Islander people about their medicines / health takes up too much time	34	1.82 (0.76)	1.59 (0.61)	**0.044**
I am not paid to work with Aboriginal and Torres Strait Islander people in relation to their health	34	1.79 (0.77)	1.59 (0.70)	0.147
I don’t know where to refer people to if there is a problem	33	2.45 (1.03)	1.79 (0.74)	**< 0.001**
Pharmacists are not ready to take up new culturally responsive roles in patient-centred care	34	1.59 (0.70)	1.47 (0.51)	0.353

^a^Not all participants answered each question. Lower scores indicate reduced perception of barriers

Most significant improvements were observed for motivators related to the pharmacist's role in improving health outcomes of Indigenous people, reduction in barriers related to having dedicated time, knowing how to talk with Indigenous people, the availability of self-help resources and knowing where to refer in case of problems.

## Discussion

Overall, this study achieved the aims of developing and evaluating a cross-cultural training programme to enable community pharmacists to deliver a culturally responsive medication review intervention for Indigenous Australians.

The programme was developed through an iterative process involving key stakeholders and end-users, testing initial content and outcome measures in a small pilot before finalising programme development for the feasibility evaluation. The feasibility testing of the training programme was undertaken as planned, with positive outcomes suggesting that the multifaceted cross-cultural training was feasible and acceptable to pharmacist participants. The content, structure and presentation of the programme were well accepted in the quantitative measures, and positive comments were made by participants in their qualitative responses.

The quantitative pre-post measures demonstrated positive changes in participants’ self-rated cultural capability, confidence and skills, motivators and barriers in relation to Indigenous health and wellbeing and working with Indigenous Australians. In particular, participants showed increased confidence in communicating with Indigenous patients about their medicine use and other health-related problems and increased motivation to improve health care for Indigenous Australians.

Whilst positive changes in capability, skills and confidence were reflected in participants’ reduced perceptions of barriers and improved motivation for working with Indigenous Australians, cultural capability areas that could be improved were also identified. This reiterates the importance of evaluating the second and third stages of the training framework outlined previously, which were implemented during the intervention delivery phase of the IMeRSe feasibility study and which will be conducted at study conclusion. Support for the importance of such a staged approach in training/education, followed by cultural mentoring, was reported recently in a study with Australian general practice trainees; baseline learning in the form of cultural education which prepared trainees for clinical practice and ongoing cultural mentoring was found to be important for longitudinal, relationship-based, learning [40].

A unique aspect of our feasibility evaluation was including an outcome measure to assess self-rated capability and confidence (the Cultural Capability Measurement Tool [35, 36]) that was developed and validated in an Indigenous, Australian-led, context with health students. This will allow future comparisons of self-rated outcomes in other health professional and health student studies.

With regard to the training development process in this study, other unique aspects were as follows: (i) using the *Stay Strong App*, a tool specifically developed for use with Indigenous Australians to guide the medicines review as part of a three-way conversation and relationship-building processes (between the pharmacist, patient and Indigenous health worker), whereby each party has the opportunity to share and learn from others, and (ii) using the *Stay Strong App* in the filmed role-plays of real-life scenarios highlighting cultural, communication and relationship-building strategies which participants watched, critiqued and then practiced skills they had observed in interactive role-play groups. As far as we are aware, this is the first time that the *Stay Strong App* has been used to identify and solve medication-related problems for Indigenous Australians. This approach aligns well with recommendations from the most recent systematic review of cultural competency interventions, which emphasises teaching practical skills to the health workforce [7]. This same systematic review also recommended that health practitioners needed to consistently apply these skills in their healthcare encounters. Pharmacist practitioners participating in the IMeRSe feasibility study were encouraged to utilise the *Stay Strong App* as an integral part of intervention delivery. Assessment of use, including rates, and qualitative acceptability with this tool as a communication and relationship-building aid for pharmacists, Indigenous health workers and Indigenous patients, is a focus of the IMeRSe feasibility study [23].

There are several limitations to this study. Firstly, due to the pre-post design, we cannot confirm that the positive changes in capability, skills and confidence are solely attributable to the cross-cultural training programme. Secondly, the sample size was small and there may have been selection bias amongst the pharmacists who participated, which limits the generalisability of findings. Additionally, whilst we attempted to ensure questionnaire responses were anonymous, with participants generating their own identification numbers (based on a combination of letters from their name and digits from date of birth), the small sample size may had led to participants recording socially desirable responses. Participants may also have had a pre-existing interest in working with Indigenous Australians and possibly more positive attitudes and increased skills and confidence given that their community pharmacy was participating in the IMeRSe feasibility study. That said, only a quarter of participants reported that they had completed any form of cross-cultural training previously. Thirdly, we conducted four workshops between March and November 2018 to accommodate staggered study implementation dates across the nine IMeRSe study sites and to facilitate small training groups at each workshop. This meant that the participant group for each workshop was small, and hence, comparison between the sites was not attempted. However, the standardised training framework, supported by online and manualised resources, ensured consistency in content and delivery across all four training workshops and scalable future implementation.

## Conclusion

This paper provides preliminary evidence that the cross-cultural training programme developed as an essential component of the IMeRSe study was acceptable and feasible, preparing community pharmacists to facilitate delivery of a culturally responsive medication review intervention for Indigenous Australians in urban centres or remote communities. The training development process combined key elements of the Aboriginal and Torres Strait Islander Health Curriculum Framework with practical application of skills using tools and resources that reflected a holistic, strengths-based focus. The multifaceted programme included a standardised online module and face-to-face workshop delivered alongside a training manual which can be operationalised for larger-scale implementation of the intervention.

We recognise that culturally responsive health workforce development encompasses more than the training programme described here, nonetheless, this study provides support for cross-cultural training, as a critical element in the implementation of culturally safe health services for Indigenous people.

## Data Availability

The dataset generated and analysed for this study is not publicly available as consent from participants was not sought to share the data more widely than for the purpose of this study.

## Footnotes

1 Please note that the use of the term ‘Indigenous’ in this manuscript includes all Aboriginal and Torres Strait Islander people and acknowledges their rich traditions and heterogenous cultures.

2 The MedsCheck and Diabetes MedsCheck Program provide for in-pharmacy medicine use reviews for consumers taking multiple medications and/or have newly diagnosed or poorly controlled Type 2 diabetes. These reviews are aimed at enhancing the quality use of medicines and reducing the number of adverse drug events experienced by consumers.

3 A Home Medicines Review (HMR) is designed to enhance the quality use of medicines, and reduce the number of adverse medicine events, by assisting consumers to better manage and understand their medicines through a more in-depth medication review conducted by an accredited pharmacist in the consumer’s home. General practitioners refer people for HMRs and receive written reports from the accredited pharmacist (www. 6cpa.com.au/medication-management-programs).

4 A paper-based version was available for participants who preferred this modality.
